# The complete mitogenome of red-collared lorikeet (*Trichoglossus rubritorquis*) and its phylogenetic analysis

**DOI:** 10.1080/23802359.2019.1667917

**Published:** 2019-09-19

**Authors:** Nan Xu, Qingzheng Zhang, Rong Chen, Hongyi Liu

**Affiliations:** a College of Biology and the Environment, Nanjing Forestry University, Nanjing, China;; b Nanjing Hongshan Forest Zoo, Nanjing, China

**Keywords:** *Trichoglossus rubritorquis*, lorikeet, Psittaciformes, mitogenome, phylogenetic analysis

## Abstract

The complete mitogenome of a lorikeet, *Trichoglossus rubritorquis* (Psittaciformes, Loriidae), was determined first in the genus *Trichoglossus*. The assembled mitogenome was 17,915 bp and composed of 13 protein-coding genes, 22 tRNAs, two rRNAs and two control regions. Nucleotide composition of *T. rubritorquis* mitogenome was 30.20% A, 33.30% C, 14.04% G, and 22.46% T, with an A + T bias of 52.66%. ML tree based on 13 protein-coding genes indicated that, *Melopsittacus undulatus* of the family Psittacidae was the closest related species to *T. rubritorquis*. This result suggested that lorikeets might still be in the family Psittacidae.

It is widely accepted that the order Psittaciformes comprises nearly 400 species, including parrots (Psittacidae), cockatoos (Cacatuidae) and lorikeets (Loriidae) (Astuti et al. 2006; Liu et al. 2019a). The red-collared lorikeet (*Trichoglossus rubritorquis*) belongs to the genus *Trichoglossus* in the family Loriidae (Wicks and Schultz 2008; BirdLife International 2016; Astuti et al. 2006). This species is only distributed in north Australia, and its native habitat is forest, shrubland and wetland (BirdLife International 2016). The population of red-collared lorikeet is suspected to be in decline due to illegal harvesting for the pet trade. It has been listed on CITES Appendix II since 1981 (BirdLife International 2016). To our knowledge, there is no previous report on the complete mitochondrial genome sequences of *T. rubritorquis* or other species in the genus *Trichoglossus*.

In this study, the complete mitogenome of *T. rubritorquis* was determined. The specimen was collected from Nanjing Hongshan Forest Zoo (32.09°N, 118.80°E), and kept in the zoological museum of Nanjing Forestry University (Accession HSZ201904). Genomic DNA was extracted following the traditional phenol-chloroform extraction procedure (Sambrook and Russell 2001). PCR amplification and Sanger sequencing were performed to obtain the mitochondrial genome sequences of *T. rubritorquis*. The complete mitogenome was 17,915 bp in length (GenBank accession MN182499), including 13 protein-coding genes, 22 tRNAs, two rRNAs and two control regions. The overall base composition was 30.20% A, 33.30% C, 14.04% G, and 22.46% T, with an A + T bias of 52.66%. Most components were transcribed on the heavy strand, except ND6 and 8 tRNAs (Gln, Ala, Asn, Cys, Tyr, Ser, Glu, Pro) transcribed on the light strand. Eleven protein-coding genes started with the common initiate codon ATG, while CO1 and ND3 initiated with GTG and ATT respectively. Expect for CO3 and ND4 ended with a single T, other protein-coding genes used the complete termination codons TAA or TAG. The mitogenome of *T. rubritorquis* displayed similar characteristics to those of other species in Psittaciformes (Eberhard and Wright 2016; Liu et al. 2019b).

ML tree of 25 species of Psittaciformes based on 13 protein-coding genes was reconstructed, using *Vanellus cinereus*, *Streptopelia chinensis* and *Aquila heliaca* as outgroups (Guindon et al. 2010). The phylogenetic analysis indicated that *T. rubritorquis* of the family Loriidae was closely related to *Melopsittacus undulatus* of family Psittacidae (Figure 1). The complete mitogenome obtained here could contribute to the studies on population genetics and molecular systematic of *T. rubritorquis* even the *genus Trichoglossus*.

**Figure 1.**
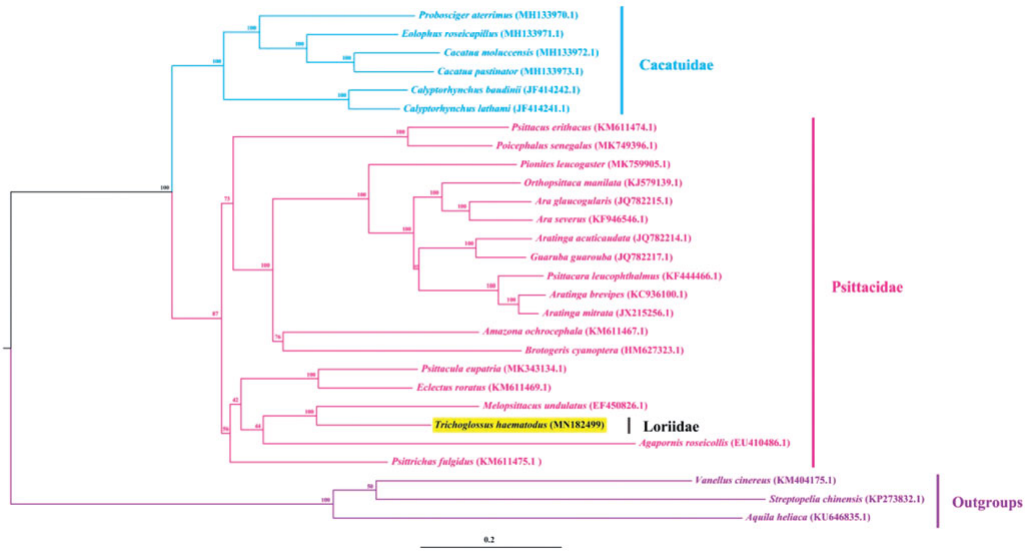
ML tree of 25 species of Psittaciformes based on 13 protein-coding genes. *V. cinereus*, *S. chinensis* and *A. heliaca* were set as outgroups. Numbers on the nodes are bootstrap support values and numbers following scientific names are GenBank accessions.
